# Investigation of the Adsorption and Reactions of Methyl Radicals on Transition Metal (M = Co, Ni, Pd, Pt) (111) Surfaces in Aqueous Suspensions

**DOI:** 10.3390/molecules30153065

**Published:** 2025-07-22

**Authors:** Pankaj Kumar, Dan Meyerstein, Amir Mizrahi, Haya Kornweitz

**Affiliations:** 1 Chemical Sciences Department, The Radical Reactions Research Center, Ariel University, Ariel 4070000, Israel; pankajkumar121091@gmail.com; 2 Chemistry Department, Ben-Gurion University, Beer-Sheva 8410501, Israel; 3 Nuclear Research Centre Negev, Beer-Sheva 84190, Israel; amirmizrachi@gmail.com

**Keywords:** DFT, CH_3_ radical, ethane production, heterogeneous reaction, transition metals

## Abstract

The DFT method was used to evaluate the adsorption of methyl radicals and the evolution of ethane on the M(111) (M = Co, Ni, Pd, Pt) surfaces, eight metal atoms, in aqueous medium. A maximum of five and four radicals can be adsorbed on Co(111) and Ni(111), respectively, and six on Pd(111) and Pt(111) (top site). The ethane evolution occurs via the Langmuir–Hinshelwood (LH) or Eley–Rideal (ER) mechanisms. The production of ethane through the interaction of two adsorbed radicals is thermodynamically feasible for high coverage ratios on the four surfaces; however, kinetically, it is feasible at room temperature only on Co(111) at a coverage of (5/5) and on Pd(111) at a coverage ratio of 4/6, 5/6, and 6/6. Ethane production occurs via the ER mechanism: a collision with solvated methyl radical produces either C_2_H_6_ or ${CH}_{2}^{*}+{CH}_{4(aq)}$. On Pd(111) the product is only C_2_H_6_, on Pt(111), both products (C_2_H_6_ or ${CH}_{2}^{*}$) are plausible, and on Co(111) and Ni(111), only ${CH}_{2}^{*}+{CH}_{4(aq)}$ is produced. Further reactions of ${CH}_{2}^{*}$ with ${CH}_{2}^{*}$ or ${CH}_{3}^{*}$ to give $C_{2}H_{4}^{*}$ or $C_{2}H_{5}^{*}$ are thermodynamically plausible only on Pt(111); however, they are very slow due to high energy barriers, 1.48 and 1.36 eV, respectively.

## 1. Introduction

In recent decades, radical chemistry has evolved into a significant and essential component of organic chemistry. Even though Gomberg [1] identified the first instance of an organic radical in 1900, the progress in the field was quite gradual for the following decades, and radicals seldom found application in synthesis [2,3]. A common structural component found in many biological substances, medications, and materials is the methyl group (CH_3_) [4,5,6,7]. A diverse range of well-known methylation agents has been created for the introduction of methyl radicals, comprising CH_3_OH, DMSO, CH_4_, and HC(OCH_3_)_3_, along with a variety of peroxides like *t*-BuOOH (TBHP), *t*-BuOOCOPh (TBPO), dicumyl peroxide (DCP), cumyl hydroperoxide (CRHP), and *t*-BuOOtBu (DTBP), among others [8].

Radicals are often formed near surfaces, e.g., in electrochemistry (e.g., the Kolbe reaction [9,10,11], reduction of halo-organic compounds [12], etc.), photocatalytic process (e.g., on TiO_2_ [13,14,15,16,17,18] and other semiconductors [19,20], where the added electron in the conduction band reduces a variety of substrates and the hole in the valence band oxidizes a variety of substrates), heterogeneous catalytic processes, e.g., catalytic hydrogenations and de-hydrogenations on Pt^0^ [21], M^0^-NPs catalysed de-halogenations by ${BH}_{4}^{-}$ [22,23,24], heterogeneous catalysed Fenton-like processes [25,26], and photo-Fenton-like processes [27]. The radicals thus formed often react with the surfaces, if not formed bound to the surface, as the reactions of radicals with metals and semiconductors are fast [28].

Reactions of methyl radicals are of importance, as discussed above. Furthermore, their reactions are considered as models for the reactions of other alkyl radicals, though the bond strengths of the products formed often differ considerably. The easiest way to study the reactions of methyl radicals is to produce them radiolytically. Methyl radicals generated radiolytically in aqueous solutions effectively react with Cr^0^, Mn^0^, Fe^0^, Ni^0^, Cu^0^, and Zn^0^ powders submerged in the solution, thus reducing the concentration of radicals and diminishing the steady state concentrations of radicals in the system [29,30]. The final products of methyl radicals observed in these studies contain CH_4_, C_2_H_4_, C_2_H_6_, C_3_H_6_, and C_3_H_8_ [29]. The adsorption of CH, CH_2_, CH_3_, and CH_4_ onto Ni(111) has been previously reported [31]. The CH_2_ and CH_3_ radicals were identified using a threshold ionization method [32]. The threefold hollow site is the best adsorption site for CH_3_ on Ni(111) [33,34,35,36,37,38,39,40]. There are two widely used kinetic models for surface reactions: the Langmuir−Hinshelwood (LH) model, in which two adsorbed species react, and the Eley–Rideal (ER) mechanism, in which a gas-phase or solvated species collides and reacts with a surface-adsorbed species.

The aforementioned studies have demonstrated that methyl radicals are adsorbed at the surface of a nanoparticle (NP) via a covalent bond [16,41] then, a catalytic dimerization occurs, according to the LH mechanism, resulting in the production of ethane [15,21,42,43]. This process is described in Equations (1)–(3):

(1) $$\left(NP\right)+{CH}_{3}^{.}\to \left(NP\right)-{CH}_{3}$$

(2) $$\left(NP\right)-{\left({CH}_{3}\right)}_{n-1}+{CH}_{3}^{.}\to \left(NP\right)-{\left({CH}_{3}\right)}_{n}$$

(3) $$\left(NP\right)-{\left({CH}_{3}\right)}_{n}\to \left(NP\right)-{\left({CH}_{3}\right)}_{n-2}+C_{2}H_{6}$$

Another mechanism for the production of ethane is via the ER mechanism, reaction 4:

(4) $${(M}^{0}-NPs)-{\left({CH}_{3}\right)}_{n}+{CH}_{3}^{.}\to {(M}^{0}-NPs)-{\left({CH}_{3}\right)}_{n-1}+C_{2}H_{6}$$

The experimental outcomes supported reaction 3 but did not eliminate the possibility of reaction 4. However, a reaction analogous to reaction 4, though in homogeneous solutions, was reported [44].

In this article, we investigate adsorption energy, charge transfer (CT), and activation energy barriers of methyl radicals on M(111) (M = Co, Ni, Pd, Pt) surfaces using density functional theory. We mainly focus on the production of ethane either by two adsorbed methyls (LH mechanism) or by one adsorbed methyl and one solvated methyl (ER mechanism), but other products are also considered. We previously investigated these processes on Cu(111), Ag(111), and Au(111) surfaces [45]. In the earlier study, the development of ethane through the LH mechanism is thermodynamically feasible for all three surfaces (Cu(111), Ag(111), Au(111)), yet kinetically, at room temperature, ethane is produced solely on Au(111) and Ag(111) under full coverage. Ethane can also be produced on Au(111) and Ag(111) via the ER mechanism in a barrierless process. On Cu(111), the products of the ER mechanism are CH_4_(aq) and an adsorbed CH_2_, which reacts further with a non-adsorbed water molecule, producing adsorbed CH_3_OH [45].

## 2. Result Analysis

### 2.1. The Adsorption of Methyl Radicals on M(111) Surfaces

**Figure 1:**
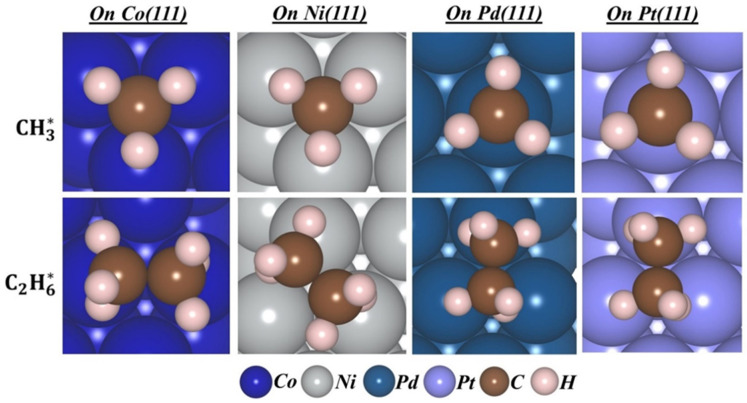
Optimized geometries, at the best adsorption site of adsorbed methyl radicals and ethane on different M(111) surfaces.

The energetically best adsorption site for the methyl radical (CH_3_·) and ethane (C_2_H_6_) on M(111) (M = Co, Ni, Pd, Pt) surfaces in aqueous solution was determined. The metallic surfaces consisted of eight metal atoms per layer. In this section, the adsorption of one methyl radical is considered. Figure 1 illustrates the optimized ground state geometries of the methyl radical and ethane molecule adsorbed on M(111) surfaces. Table 1 presents the adsorption energies, CT, and binding distances. The best adsorption site for methyl radicals in the aqueous suspensions is the fcc site for Co(111) and Ni(111) surfaces, as was reported previously [33,34,35,36,37,38,39,40], while for Pd(111) and Pt(111) surfaces, it is the atop site. The best adsorption site for ethane is the hcp site for Ni(111), Pd(111), and Pt(111) surfaces, while for the Co(111) surface, it is the bridge site.

The determined values of the nearest binding distances, CT, and adsorption energies of CH_3_ and C_2_H_6_ adsorbed to M(111) surfaces in aqueous suspensions are presented in Table 1. The optimal adsorption of CH_3_ shows a gradual decline in the following order: Co(111) > Pt(111) > Ni(111) > Pd(111), whereas for C_2_H_6_, the decreasing trend is in the order of Pt(111) > Pd(111) > Ni(111) > Co(111). Tables S1 and S2 present the values for ZPE and adsorption energies of methyl radicals and ethane at optimal adsorption locations. The highest CT from surface to CH_3_ is observed at the Co(111) surface (0.40 e), and then on Ni(111) (0.37 e), whereas the lowest CT to CH_3_ occurs on Pt(111) (0.04 e), while on Pd, the value is similar (0.07 e). For ethane, the CT is very small, on Co and Ni the CT is from the surface to the molecule (0.01 and 0.03 e, respectively), while for Pd and Pt, surfaces act as an acceptor, exhibiting small negative values of CT (−0.02 and −0.04 e, respectively), meaning CT from molecule to surface. Tables S3 and S4 present the CT values for methyl radicals and ethane across all adsorption sites in the aqueous phase. The binding distances between M(111) and C atoms are similar for CH_3_ across all metals, whereas for C_2_H_6_, the closest interaction is observed on Pd(111). All the optimized structures of methyl radicals and ethane on M(111) surfaces at all adsorption sites are depicted in Table S5 for methyl radicals and in Table S6 for ethane. The nearest binding distances for various numbers of methyl radicals adsorbed on M(111) surfaces are depicted in Figure S1.

### 2.2. Adsorption of Methyl Radicals at Higher Coverage

**Figure 2:**
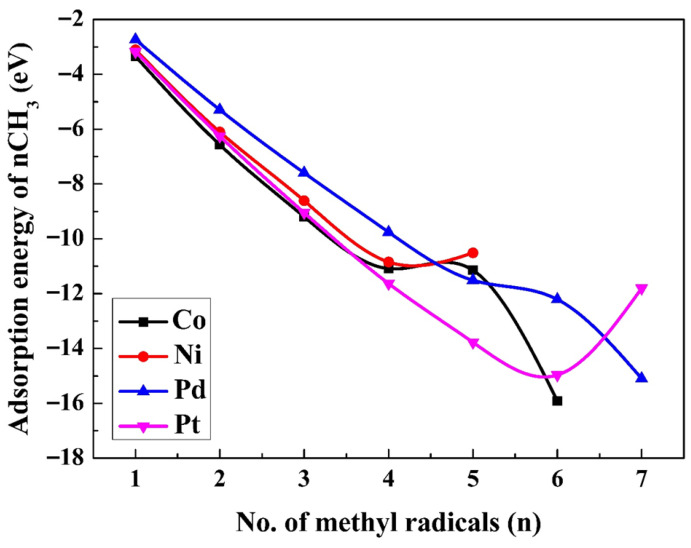
Adsorption energies ($E_{ads}$) for n methyl radicals on the M(111) surfaces in an aqueous phase (using Equation (5)).

Figure 2 shows the total adsorption energies for n (where n = 1–6 for Co; n = 1–7 for Pd and Pt, and n = 1–5 for Ni) methyl radicals on the four M(111) surfaces. Table S7 displays all of the optimal structures for methyl radicals on the M(111) surfaces. As depicted in Figure 2, Ni and Pt surfaces show a monotonic decreasing trend Ni and Pt surfaces show a monotonic decreasing trend up to four (Ni) and six (Pt) methyls, and then, the value rises, suggesting that the adsorption of the fifth and seventh methyl radicals is unfavoured. For Co and Pd, there is a twist in the graph, indicating that the structures with six Co(111) and seven Pd(111) methyl radicals are distorted. The addition of another methyl radical onto a surface containing n − 1 (n = 1–7) pre-adsorbed methyl radicals result in n adsorbed radicals. The adsorption energy of the last methyl is given in Table S8. Reaction 5 was used to assess the thermodynamic limit of the coverage ratio. The free energy (${∆G}_{{CH}_{3}(aq)}(5)$) for reaction 5 was determined for each surface:

(5) $${(n-1)CH}_{3}^{*}+{CH}_{3(aq)}^{.}\to {nCH}_{3}^{*}$$

**Figure 3:**
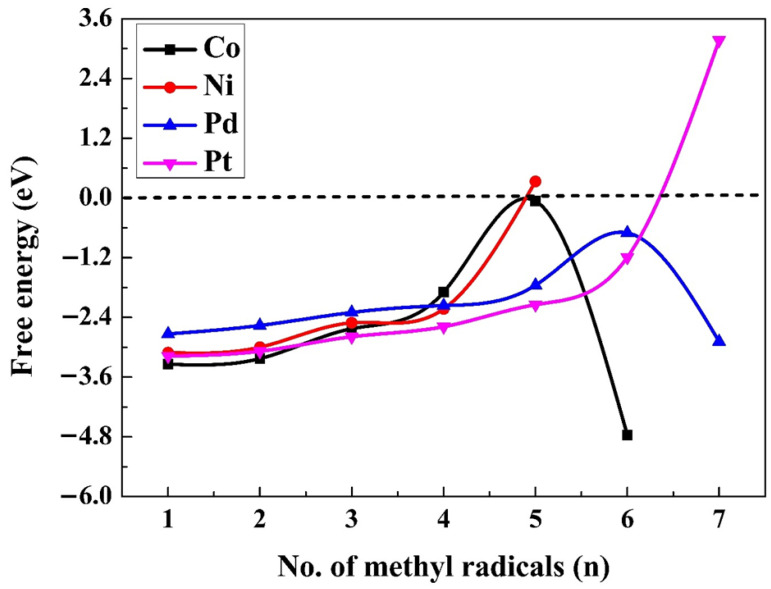
Gibbs free energies (${∆G}_{{CH}_{3}ads}$) for the adsorption of an additional methyl radical on the M(111) surfaces (using Equation (5)).

The values of ${∆G}_{{CH}_{3}\left(aq\right)}(5)$ on the fcc hollow sites of Co(111) and Ni(111) surfaces, and at the top positions on the Pd(111) and Pt(111) surfaces, are gradually enlarged by sequentially adding one methyl radical at the closest fcc or top sites. The process of adding adsorbed methyl radicals persisted until the free energy of adsorption (${∆G}_{{CH}_{3}(aq)}(5)$) for reaction 5 turned endergonic on Ni(111), Co(111), Pd(111), and Pt(111). No more than five radicals can be adsorbed on Co, because for six methyl radicals the structure is completely distorted as depicted in Table S7, for the fifth radical, the free energy of adsorption becomes almost zero (−0.06 eV). On Ni(111), only four CH_3_ radicals are adsorbed spontaneously; the adsorption of the fifth methyl is endergonic. On Pd(111) and Pt(111), six methyl radicals can be adsorbed. For Pt, the adsorption of the seventh radical is endergonic; therefore, no more than six methyl radicals can be adsorbed spontaneously, while for Pd, the adsorption of the seventh radical distorts the structure, as depicted in Table S8; therefore, the adsorption of only six radicals is considered. The ${∆G}_{{CH}_{3}\left(aq\right)}(5)$ values are presented in Figure 3. The charge transfer for all the adsorbed methyl radicals is presented in Figures S2 and S3, respectively.

### 2.3. Projected Density of States

**Figure 4:**
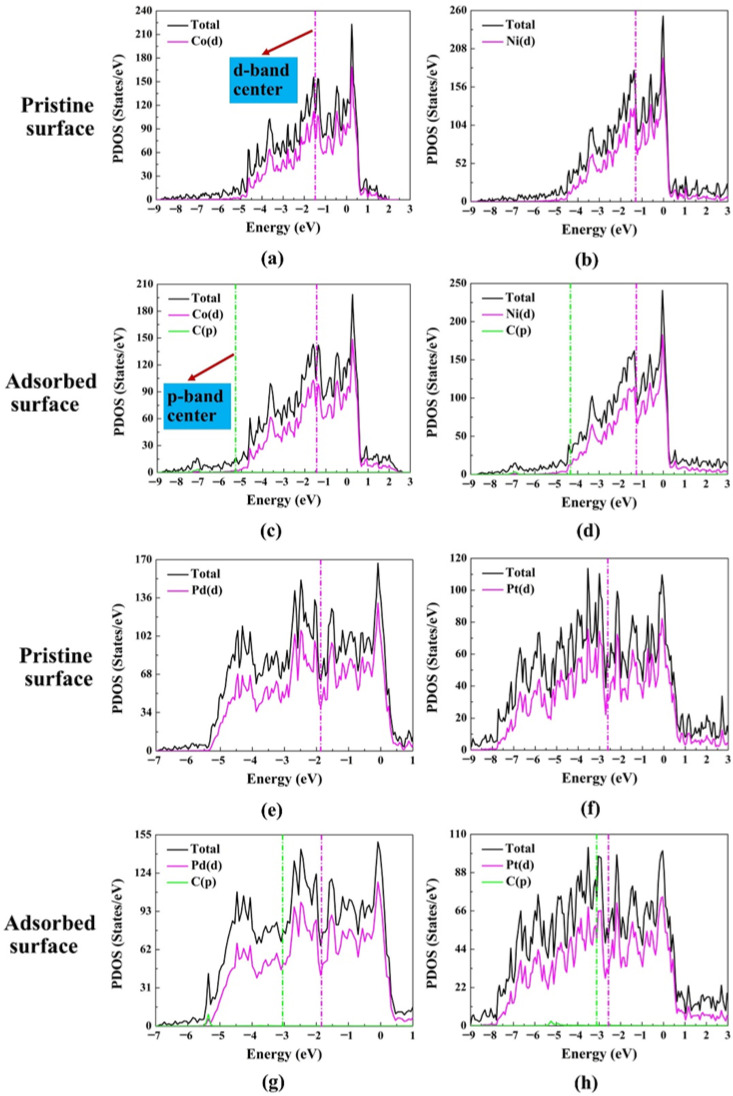
The projected density of states for a pristine M(111) surface, M = Co, Ni, Pd, and Pt (**a**,**b**,**e**,**f**), and of the surfaces with an adsorbed CH_3_ (**c**,**d**,**g**,**h**).

The electronic properties of M(111) surfaces from the perspective of the electronic projected density of state are shown in Figure 4. Figure 4 displays the contribution of the projected density of states (PDOS) orbitals around the Fermi level of the M(111) surfaces. Most of the theoretical studies based on first principles concentrate on understanding how the adsorbate interacts with the d-electrons of the surfaces of transition metals [46,47,48,49,50,51]. The d-band center model created by Hammer and Nørskov over a decade ago is the most commonly used framework to analyze the function of d-electrons [52,53,54,55]. The d-band center ($\varepsilon_{d}$) and p-band center ($\varepsilon_{p}$) are determined through the equation provided below [56]:

(6) $$\varepsilon_{x}=\frac{\int_{\varepsilon_{min}}^{\varepsilon_{max}}n_{x}(\varepsilon )\varepsilon d\varepsilon }{\int_{\varepsilon_{min}}^{\varepsilon_{max}}n_{x}(\varepsilon )d\varepsilon }$$

The band center for each orbital (x = p, d) is denoted by $\varepsilon_{x}$, which corresponds to the p-band center ($\varepsilon_{p}$) and d-band center ($\varepsilon_{d}$).

Figure 4 {(a, c), (b, d), (e, g) and (f, h)} presents the projected density of states for pristine metallic surface and a surface with an adsorbed CH_3_ of Co(111), Ni(111), Pd(111) and Pt(111), respectively. The purple vertical line represents the d-band center value in all the figures in the diagram of PDOS, and the green vertical line represents the pbc of the carbon of the adsorbed methyl. The values of the dbc and the pbc are given in Table 2. For all the metals, the dbc for the pristine metal and the dbc for the metal with the adsorbate are almost the same; only a small shift to a higher value is observed (0.02–0.04 eV). In principle, as the dbc is higher, the adsorption should be stronger, as fewer anti-bonding states are occupied; therefore, lower adsorption energies are expected on Co(111) and Ni(111) than on Pd(111) and Pt(111). Surprisingly, the adsorption energy on Pt(111) is very low (−3.18 eV). The reason is the minimal value (0.50 eV) of the dbc-pbc, as depicted in Table 2, indicating a very good overlap between the d-band of the metal and the p-band of the carbon (C) of the adsorbed CH_3_. In addition, Co(111) has a lower dbc (−1.47 eV) than Ni(111) (−1.30 eV), but the adsorption energy is stronger (−3.34 eV) than on Ni(111) (−3.11 eV). The strong adsorption on Co(111) is attributed to the smaller valence electrons (9) than all the other metals (10); therefore, fewer electrons are available in the case of Co(111) to occupy the anti-bonding states.

### 2.4. Production of Ethane via the LH Mechanism—A Reaction of Two out of n Adsorbed Radicals

In this section, we examine the ethane evolution reaction at various coverage ratios up to 5/5 for Co(111), 6/6 for Pd(111) and Pt(111) surfaces, as well as 4/4 for the Ni(111) surface, according to the adsorption ratios observed in Section 2.2. The free energies of ethane evolution via the LH mechanism ($∆G_{C_{2}H_{6}\left(aq\right)}^{0}(7)$) are determined through reaction 7:

(7) $${nCH}_{3}^{*}\to {(n-2)CH}_{3}^{*}+C_{2}H_{6(aq)}$$

**Figure 5:**
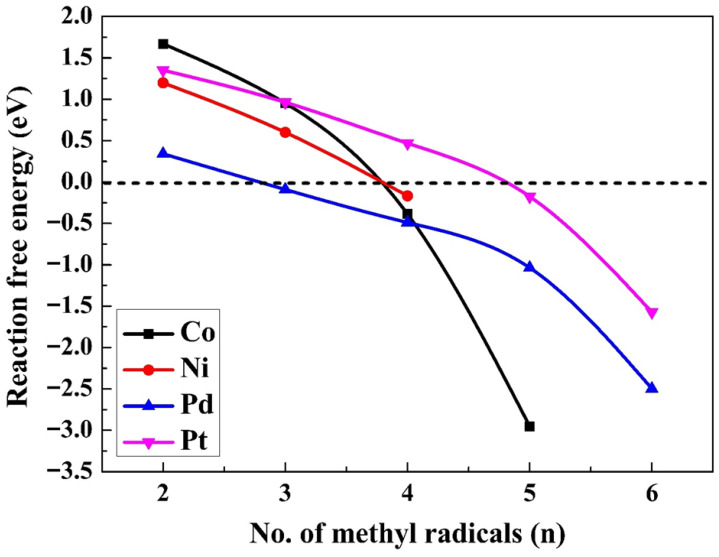
Reaction-free energies (${∆G}^{0}$) for the evolution of ethane on the M(111) surfaces (using Equation (7)). The dotted line indicates zero energy.

The standard reaction free energies for ethane formation ($∆G_{C_{2}H_{6}\left(aq\right)}^{0}(7)$), based on reaction 7, are presented in Figure 5 for the aqueous suspensions. $∆G_{C_{2}H_{6}\left(aq\right)}^{0}$ values and barrier heights for n = 6 (Pd, Pt), n = 5 (Co, Pd, Pt), and n = 4 (Co, Ni, Pd) are given in Table 3. Based on the findings in Table S9, ethane will not be formed on all M(111) surfaces with a coverage ratio of 2/n. Since the adsorption energy diminishes as the coverage ratio increases (Figure 3), the feasibility of ethane formation increases. Based on the findings shown in Figure 5, the highest exergonicity for this reaction is seen on Pd(111), followed by Co(111), while the values for Ni(111) and Pt(111) are considerably smaller. The exergonicity increases in magnitude (becomes more negative) with a rise in the coverage ratio. These values diminish from 1 to n (n = 5 for Co, 6 for Pd and Pt, and 4 for Ni), with Co(111) changing from 1.67 to −2.95 eV, Ni(111) from 1.20 to −0.17 eV, Pd(111) from 0.34 to −2.50 eV, and Pt(111) from 1.35 to −1.57 eV. All the reaction free energies ΔG^0^(7) for the production of ethane on various M^0^(111) surfaces at different surface coverage ratios are provided in Table S9.

According to the results presented in Table 3, the lowest barrier was found for Pd at high coverage (n = 6) (0.41 eV), then on Co at high coverage (n = 5) (0.52 eV). Moderate barriers were observed for Pd; n = 5 (0.65 eV) and n = 4 (0.83 eV). All other barriers are very high and exclude the formation of ethane via this mechanism. Table 3 also presents the carbon–carbon bond distances in both the initial and the transition states.

**Figure 6:**
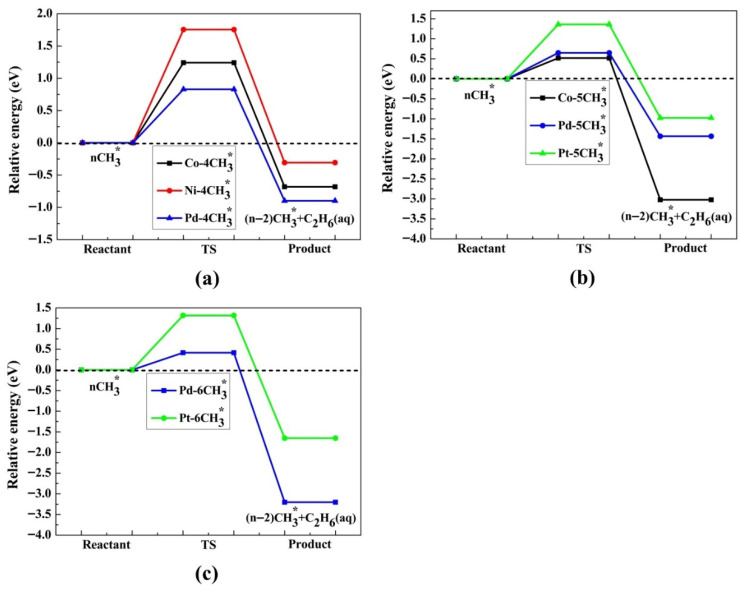
The activation energy barrier (E_a_) for the evolution of ethane in the case of (**a**) 4CH_3_ for Co, Ni, Pd; (**b**) 5CH_3_ for Co, Pd, Pt; (**c**) 6CH_3_ for Pd, Pt. The dotted line indicates zero energy.

**Figure 7:**
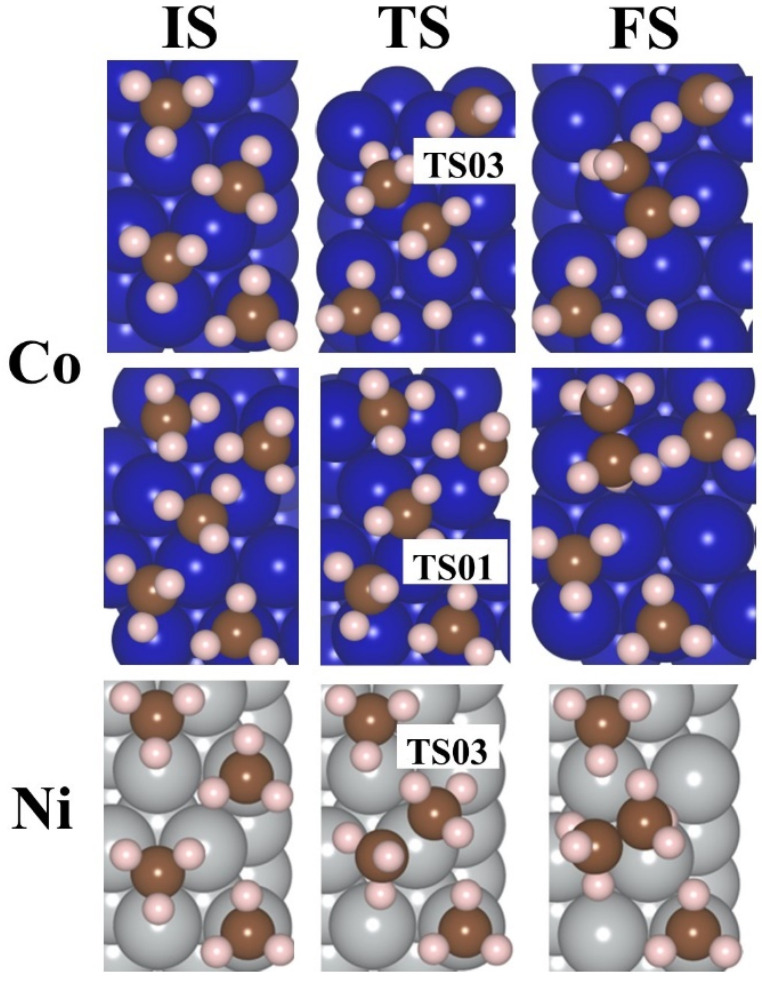
The IS, TS, and FS for 4–5CH_3_ are placed on the Co(111) and Ni(111) surfaces for evolution of ethane. Dark blue—Co, gray—Ni, brown—C, and pink—H.

**Figure 8:**
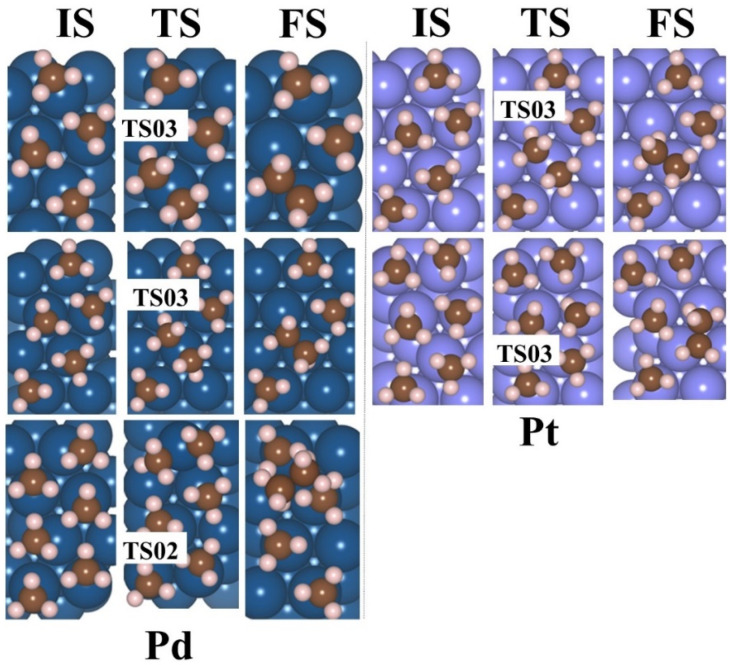
The IS, TS, and FS of 5–6CH_3_ for Pd(111) and Pt(111) surfaces for the evolution of ethane. blue—Pd, light blue—Pt, brown—C, and pink—H.

Figure 6 illustrates the lowest barrier alongside the exergonic reaction, on M(111) surfaces, for n = 4, 5, 6. Additionally, the activation barrier for n = 3–6 CH_3_ adsorbed on Pd(111) is presented in Figure S4. The activation energy barrier is lowered with an increase in the coverage ratio; for Co(111), it reduces from 1.24 to 0.52 eV (4–5 CH_3_), for Ni(111) it is 1.75 eV (4 CH_3_), for Pd(111) it decreases from 1.19 to 0.41 eV (3–6 CH_3_), and for Pt(111) it is 1.36 to 1.32 eV (5–6 CH_3_). These values suggest that the production of ethane via this mechanism is feasible exclusively on the Pd(111) and Co(111) surfaces. As the coverage ratio rises, the activation energy barrier values are shown in Table S10. The structures for the initial state (IS), TS, and final state (FS) are provided in Figure 7 and Figure 8 for 4–6 CH_3_ adsorbed on M(111) surfaces, respectively.

### 2.5. Ethane Production via the RE Mechanism—Reaction Between One Adsorbed Methyl Radical and Another One in Solution


(8) $${CH}_{3}^{*}+{CH}_{3(aq)}^{.}\to {C_{2}H}_{6}^{*}$$

(9) $${CH}_{3}^{*}+{CH}_{3(aq)}^{.}\to {CH}_{2}^{*}+{CH}_{4(aq)}$$

(10) $${CH}_{2}^{*}+{CH}_{4(aq)}\to C_{2}H_{6}^{*}$$

**Figure 9:**
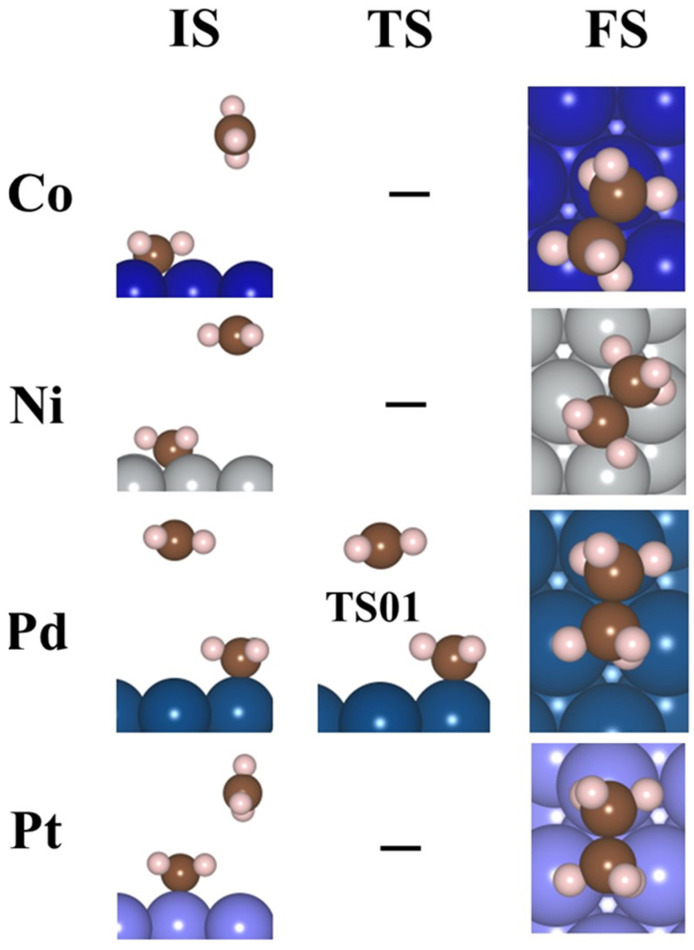
The IS, TS, and FS for the evolution of ethane according to reaction 8. dark blue—Co, grey—Ni, blue—Pd, light blue—Pt, brown—C, and pink—H.

The generation of ethane is examined via the ER mechanism—a methyl radical moves randomly within the aqueous solution, and while moving, it collides with an adsorbed methyl, and ethane is produced according to reaction 8 or methane according to reaction 9 followed by reaction 10. Reaction 8 and reaction 9 are competing reactions, constrained by the lifetime of the ${CH}_{3(aq)}$ radical in solution. Both reactions may occur on Pt(111), Ni(111), and Co(111), while on Pd(111), only reaction 8 occurs, and ${CH}_{2}^{*}$ is not formed according to reaction 9. Table 4 provides the reaction free energies and barriers for these reactions. The IS, TS, and FS figures are shown in Figure 9. For Pt(111), both reactions are barrierless; therefore, ethane and ${CH}_{2}^{*}$ are both produced via the ER mechanism. On Ni(111) and Co(111), reaction 9 is barrierless, while there is a barrier for reaction 8 (0.16 eV and 0.56 eV, respectively); therefore, reaction 9 is predominant, and ${CH}_{2}^{*}$ is produced. A small barrier is also observed for reaction 8 on Pd(111) (0.21 eV). The small barrier on Ni(111) and Pd(111) enables reaction 8 to occur if the steady state concentration of ${CH}_{3}^{.}$ is small, e.g., in continuous photolysis or radiolysis. Formation of ethane via the RE mechanism on Co(111) is less likely.

The formation of ${CH}_{2}^{*}$ on Ni(111) was reported previously [31]. On Co(111) and Ni(111), reaction 10 is endergonic (0.82 and 0.52 eV, respectively), preventing the production of ethane via this mechanism. On Pt(111), reaction 10 is exergonic (−0.03 eV), but $C_{2}H_{6}$ is not formed via reaction 10 as the barrier for this reaction is very high, 1.60 eV.

Reaction of ${CH}_{2}^{*}$ and ${CH}_{3}^{*}$ to form $C_{2}H_{4}^{*}$ and $C_{2}H_{5}^{*}$ on M(111) surfaces:

(11) $${CH}_{2}^{*}+{CH}_{2}^{*}\to C_{2}H_{4}^{*}$$

(12) $${CH}_{2}^{*}+{CH}_{3}^{*}\to C_{2}H_{5}^{*}$$

Reactions 11 and 12 do not occur on Co(111) and Ni(111) surfaces as they are endergonic (for Co(111), ΔG^0^ = 0.48 eV (reaction 11) and 0.67 eV (reaction 12); for Ni(111), the values are 0.13 and 0.72 eV, respectively); however, these reactions may occur on Pt(111) surfaces since they are exergonic reactions, −0.61 and −0.13 eV. These values and the values of the energy barriers are given in Table 4. These reactions are expected to be very slow due to their high barriers (1.48 eV, rate constant = 9.65 × 10^−14^ M^−1^ s^−1^ for reaction 11 and 1.36 eV, rate constant = 1.06 × 10^−11^ M^−1^ s^−1^ for reaction 12). We have assumed, based on Eyring theory [57], that the rate constant for a reaction between two radicals in aqueous solution is 10^12^. $C_{2}H_{4}^{*}$ and $C_{2}H_{5}^{*}$ are not produced on Pd(111) as ${CH}_{2}^{*}$ is not formed according to reaction 9 on this surface. The structures of IS, TS, and FS for these reactions are depicted in Tables S11–S14.

The reaction of ${CH}_{2}^{*}$ with ${CH}_{3(aq)}^{.}$ according to the RE mechanism was studied as well. This reaction does not occur directly, ${CH}_{3(aq)}^{.}$ is first adsorbed, and only then, it reacts with ${CH}_{2}^{*}$, this is an exergonic reaction only on Pt(111) (−3.31 eV). The high adsorption energy of ${CH}_{3(aq)}^{.}$ on Pt(111) (−3.18 eV) is enough to overcome the high barrier of reaction 12 (1.36 eV), so C_2_H_5_ is produced on Pt(111), as was determined experimentally [30]. The formation of $C_{2}H_{5}^{*}$ on Pt(111) can be followed by one of the following reactions:

(13) $$C_{2}H_{5}^{*}\to C_{2}H_{4}^{*}+H^{*}$$

(14) $$C_{2}H_{5}^{*}+{CH}_{3}^{*}\to C_{2}H_{4}^{*}+{CH}_{4(aq)}$$

(15) $$C_{2}H_{5}^{*}+{CH}_{3}^{*}\to C_{3}H_{8}^{*}$$

According to reaction 13, $C_{2}H_{5}^{*}$ splits into $C_{2}H_{4}^{*}+H^{*}.$ This is an exergonic reaction (ΔG^0^ = −0.43 eV) with a barrier of 0.65 eV; $C_{2}H_{4}^{*}$ was observed as a product of the reaction of ${CH}_{3(aq)}^{.}$ with Pt^0^ [29,31].

**Figure 10:**
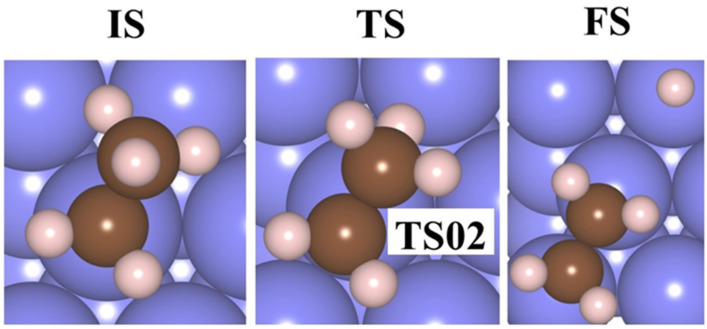
The IS, TS, and FS of $C_{2}H_{5}^{*}$ splits into $C_{2}H_{4}^{*}+H^{*}$ in solution for Pt(111) surface. light blue—Pt, brown—C, and pink—H.

Reaction 14 is an isoenergetic reaction (ΔG^0^ = 0.00 eV) with a high barrier of 1.81 eV; therefore, this reaction is not plausible on Pt(111) surfaces. The activation barrier of reaction 13 is depicted in Figure 10.

Surprisingly, recombination of an adsorbed methyl radical with an adsorbed ethyl radical, according to reaction 15, is not plausible, as this is an endergonic reaction (ΔG^0^ = 0.51 eV).

### 2.6. Formation of Methanol (CH_3_OH) on M(111) Surfaces


(16) $${CH}_{2}^{*}+H_{2}O^{*}\to {CH}_{3}{OH}^{*}$$

The ${CH}_{2}^{*}$ produced on the M(111) (M = Co, Ni, Pt) can interact with an adsorbed water molecule to yield methanol according to reaction 16. This reaction is not feasible on all the M(111) surfaces, as it is endergonic (the values are 1.24, 0.91, and 0.58 eV for Co, Ni, and Pt, respectively). Also, the reaction of ${CH}_{3}^{*}$ with an adsorbed water molecule to form ${CH}_{3}{OH}^{*}+H^{*}$ is not feasible on all the M(111) surfaces, as it is endergonic (ΔG^0^ = 0.86 eV (Co) = 0.64 eV (Ni) = 0.32 eV (Pd) = 0.63 eV (Pt)).

### 2.7. The Diffusion of Adsorbed CH_2_ and CH_3_ on M(111) Surfaces

Since it is anticipated that the CH_2_ and CH_3_ will be randomly adsorbed at their optimal adsorption locations on the M(111) surfaces, they are expected to move on the surface until they attain the required configuration for the reaction. Consequently, the energy barrier for the diffusion of an adsorbed CH_2_ and CH_3_ from one identical site to another was determined.

The diffusion process of ${CH}_{2}^{*}$ on M(111) surfaces occurs in two steps, and two barriers are involved. On Co(111), the first step refers to the shift from hcp to fcc, and the second refers to the shift from fcc to hcp. On Ni(111), the steps are fcc to hcp and then hcp to fcc. On Pt(111), the first step is the bridge to fcc, and then fcc to bridge. On Pd(111), only one barrier is observed, for the first shift from bridge to fcc, the second shift from fcc to bridge is barrierless.

The barriers are depicted in Table 5. The values are on Co of 0.48 and 0.09 eV; on Ni of 0.23 and 0.46 eV; and Pt of 0.04 and 0.67 eV; on Pd, a barrier of 1.03 eV was found for the diffusion of ${CH}_{2}^{*}$, but ${CH}_{2}^{*}$ is not formed on Pd(111).

The diffusion of methyl radicals (${CH}_{3}^{*}$) on the surface presents a minimal barrier on Co (0.04 eV), while the barrier is higher for Ni (0.50 eV), Pd (0.28 eV), and even higher for Pt (0.65 eV).

These barriers are lower than the relevant barriers of reactions 7, 11, and 12; therefore, they are not expected to affect these reactions. The rate constants of the relevant processes are given in Table 6. The structures of the initial state (IS), TS, and final state (FS) for Co(111), Ni(111), Pd(111), and Pt(111) are presented in Tables S15, S16, S17 and S18, respectively.

In Table 6, the rate constants for the diffusion of ${CH}_{3}^{*}$ and ${CH}_{2}^{*}$ and for the formation of ethane, according to the LH mechanism (reaction 7) and RE mechanism (reaction 8) are displayed. The rate constants are calculated according to the barriers. The diffusion rate constants of ${CH}_{2}^{*}$ and ${CH}_{3}^{*}$ are higher than the rate constant for the reactions given in Table 6; therefore, the diffusion of these species does not affect the rate of these reactions.

## 3. Computational Methods

We used the Vienna ab-initio Simulation Package (VASP) [58,59], version 6.3.2, to conduct a non-spin polarized first-principles computation within the framework of density functional theory (DFT) [60]. To deal with electron–ion–core focused interfaces, the projector augmented wave (PAW) has been used [61]. We have operated the Perdew–Burke–Ernzerhof (PBE) [62,63] function to manage electron exchange and correlation in addition to the PAW technique. The Grimme’s DFT-D2 dispersion correction [64,65] was used to describe the long-range van der Waals (vdW) interactions. The solvent effect was considered using an implicit self-consistent electrolyte solvation model, VASPsol [66]. Explicit water molecules were not used, as no significant interactions are expected between the methyl radicals and the solvent (H_2_O). An energy cut-off of 500 eV was employed for each slab. A Monkhorst–Pack mesh of 6 × 6 × 1 k-point was used to sample the Brillouin zone [67]. The convergence accuracy criteria for all our calculations were 10^−3^ eVÅ^−1^ and 10^−5^ eV for forces and energy. The transition states (TS) were located using the Climbing Image Nudged Elastic Band method (Cl-NEB) [68] by considering five images for every state. We investigated the p- and d-band center values by using VASPKIT Standard Edition 1.5.1 (27 January 2024) [69].

All M(111) (M = Co, Ni, Pd, Pt) surfaces were represented by a six-layer slab; every layer is made up of eight metal atoms, and a 16 Å vacuum space is included between the slabs in the z-direction to prevent undesired interactions. The vacuum space is substituted with an aqueous medium utilizing VASPsol. The harmonic oscillator method was employed to conduct phonon calculations for every optimized structure, utilizing a step width of 0.015 Å to derive the zero-point vibration energies (ZPVE) of the system. These computations were employed to confirm that the optimized configurations represent real minimum or transition states (one extra imaginary frequency). The ZPVE values were utilized to determine the Gibbs free energy. The VESTA code [70,71,72] was used to illustrate the stable structures and the TS. The adsorption energy (*E_ads_*) of adsorbate on the M(111) surfaces is calculated using Equation (17):

(17) $$E_{ads}=G_{s}^{0*}-\left(G_{s}^{0}+G^{0*}\right)$$

(18) $$G^{0}=[E+ZPVE+(T*S)]$$

E, T, and S are electronic energy, room temperature (298.15 K), and entropy. $G_{s}^{0*}$ is the free energy of the surface with the adsorbate, $G^{0*}$ is the free energy of the M(111) surfaces, and $G_{s}^{0}$ is the free energy of the aqueous adsorbate. Negative $E_{ads}$ values mean that adsorption is favored, and vice versa. The free energy (${∆G}^{o}$) of a reaction was calculated using the equation:

(19) $${∆G}^{0}={\sum G}_{products}^{0}-{\sum G}_{reactants}^{0}$$

The amount of charge transfer (CT) of the adsorbate on M(111) is determined through Bader charge analysis [73,74]. Positive CT values indicate CT from the surface towards the adsorbate, whereas negative values signify CT from the adsorbate towards the surface. We determined the rate constants (k) through the application of the Arrhenius equation [75],

(20) $$k={Ae}^{-E_{a}/k_{B}T}$$

where $E_{a}$ is the activation energy barrier, *T* is the absolute room temperature (298.15 K), $k_{B}$ is the Boltzmann constant, and *A* is the pre-exponential factor. In this context, we have utilized the pre-exponential factor (*A*) value of 10^12^ to determine the rate constant. In this research, pre-adsorbed water molecules are not considered.

## 4. Concluding Remarks

In summary, results of an in-depth investigation of the properties of methyl radicals at the M(111) (M = Co, Ni, Pd, Pt) surfaces, using the density functional approach, were obtained. The adsorption of methyl radicals on M(111) surfaces, consisting of eight atoms, was explored, revealing that up to five methyl radicals can be adsorbed on Co(111), while on Pd(111) and Pt(111) surfaces up to six, and on Ni(111), only four radicals can be adsorbed. The best adsorption sites are fcc (Co and Ni) and atop (Pd and Pt). Production of ethane via the LH mechanism is endergonic on all metals for two adsorbed methyl radicals. The production of ethane is exergonic for high coverage ratios. Kinetically, it is produced at room temperature on Co at a high coverage ratio (5/5) with a barrier of 0.52 eV, and on Pd at a coverage ratio of 4/6, 5/6, and 6/6 with barriers of 0.83, 0.65, and 0.41 eV, respectively. Ethane can also be produced via the RE mechanism, one ${CH}_{3(aq)}^{.}$ radical that is moving randomly in the aqueous solution hits an adsorbed CH_3_ radical on the surface. In this scenario, the products are either ethane or ${CH}_{2}^{*}$ and ${CH}_{4(aq)}$. On Pd(111), such a collision produces only $C_{2}H_{6}$. On Ni(111) and Co(111) surfaces only production of ${CH}_{2}^{*}$ + ${CH}_{4(aq)}$ is observed without a barrier, while on Pt(111), both processes are observed; neither has a barrier, and, therefore, both are plausible. The reaction of two ${CH}_{2}^{*}$ radicals to produce $C_{2}H_{4}^{*}$ is exergonic on Pt(111), but is very slow due to its large barrier (1.48 eV). In addition, the reaction of ${CH}_{2}^{*}$ with ${CH}_{3}^{*}$ to produce $C_{2}H_{5}^{*}$ is exergonic on Pt(111) with a similar barrier (1.36 eV).

The diffusion of the ${CH}_{2}^{*}$ on the M(111) surfaces has a moderate barrier on Co, Ni, and Pt (0.48 eV, 0.46 eV, 0.67 eV, respectively), the barrier for Pd (1.03 eV) is significantly higher, but ${CH}_{2}^{*}$ is not formed on Pd(111) via the reaction of methyl radicals. The diffusion of the methyl radicals on the surface is almost barrierless for Co (0.04 eV), and moderate for the other surfaces, 0.50, 0.28, and 0.65 eV for Ni(111), Pd(111), and Pt(111), respectively. These barriers are lower than the relevant barriers of reactions 7, 11, and 12; the diffusion is faster than the reaction of these intermediates on the surface, therefore, they are not expected to affect these reactions. $C_{2}H_{5}^{*}$ and $C_{2}H_{4}^{*}$ were detected experimentally on Pt(111) [30]. The formation of $C_{2}H_{5}^{*}$ on Pt(111) occurs via the RE mechanism in a two-step mechanism, ${CH}_{3(aq)}$ is adsorbed on the Pt(111) surface, and then it reacts with ${CH}_{2}^{*}$ to produce $C_{2}H_{5}^{*}$. This reaction is plausible due to the high adsorption energy of ${CH}_{3}^{*}$ (−3.18 eV). $C_{2}H_{4}^{*}$ is produced by the de-hydrogenation of $C_{2}H_{5}^{*}$ in an exergonic reaction (ΔG^0^ = −0.43 eV) with a barrier of 0.65 eV on Pt(111) surface. These products that are produced on Pt(111) were not found on Ag(111), Au(111), and Cu(111) surfaces [45].

## Figures and Tables

**Table 1:** Values of adsorption energies (E_ads_), CT (e), and distance between M(111) and C atoms in an aqueous medium at room temperature.

Metals	Adsorbates	Adsorption Sites	Adsorption Energy (eV)	Charge Transfer ^a^ (e)	M(111)-C Distance (Å)
Co	${CH}_{3}$	fcc	−3.34	0.40	2.03
	$C_{2}H_{6}$	bridge	−0.55	0.01	2.91
Ni	${CH}_{3}$	fcc	−3.11	0.37	2.06
	$C_{2}H_{6}$	hcp	−0.75	0.03	3.16
Pd	${CH}_{3}$	top	−2.73	0.07	2.03
	$C_{2}H_{6}$	hcp	−0.68	−0.02	2.89
Pt	${CH}_{3}$	top	−3.18	0.04	2.07
	$C_{2}H_{6}$	hcp	−0.69	−0.04	3.08

^a^ Positive value specifies CT from surface to the adsorbate, and vice versa.

**Table 2:** The estimated values of d-band center (dbc) for pristine surfaces and adsorbed CH_3_ (${CH}_{3}^{*}$), p-band center (pbc), and difference of d- and p-band centers (dbc-pbc).

Metal Surfaces	dbc (Metal) (eV)		pbc ($C^{*}$) (eV)	dbc-pbc (eV)
	Pristine Surfaces	${CH}_{3}^{*}$		
Co(111)	−1.47	−1.43	−5.31	3.88
Ni(111)	−1.30	−1.28	−4.33	3.05
Pd(111)	−1.87	−1.85	−3.06	1.21
Pt(111)	−2.62	−2.59	−3.09	0.50

**Table 3:** The evaluated reaction free energies (ΔG^0^(7)), activation energy barrier (E_a_), C-C distance in the initial state (IS), and transition state (TS) on different M(111) surfaces.

M(111) Surfaces	No. of Methyl Radicals Adsorb	${∆G}^{0}\left(7\right)$ (eV)	$E_{a}$ (eV)	C-C Bond (Å) in IS	C-C Bond (Å) in TS
Co	${4CH}_{3}^{*}$	−0.39	1.24	3.46	1.85
Ni	${4CH}_{3}^{*}$	−0.17	1.75	3.72	1.93
Pd	${4CH}_{3}^{*}$	−0.49	0.83	3.21	2.01
Co	${5CH}_{3}^{*}$	−2.95	0.52	3.21	3.15
Pd	${5CH}_{3}^{*}$	−1.04	0.65	3.00	2.04
Pt	${5CH}_{3}^{*}$	−0.18	1.36	3.05	1.93
Pd	${6CH}_{3}^{*}$	−2.50	0.41	3.09	2.48
Pt	${6CH}_{3}^{*}$	−1.57	1.32	3.09	3.03

**Table 4:** The evaluated reaction free energies (ΔG^0^) and activation energy barriers (E_a_) on M(111) surfaces for reactions 8 to 12 in aqueous suspensions.

Reaction Numbers	Reactions	Co(111) (eV)		Ni(111) (eV)		Pd(111) (eV)		Pt(111) (eV)	
		${∆G}^{0}$	$E_{a}$	${∆G}^{0}$	$E_{a}$	${∆G}^{0}$	$E_{a}$	${∆G}^{0}$	$E_{a}$
8	${CH}_{3}^{*}+{CH}_{3(aq)}^{.}\to {C_{2}H}_{6}^{*}$	−2.11	0.56	−2.36	0.16	−2.86	0.21	−2.72	NB
9	${CH}_{3}^{*}+{CH}_{3(aq)}^{.}\to {CH}_{2}^{*}+{CH}_{4(aq)}$	−2.93	NB	−2.89	NB	-	-	−2.69	NB
10	${CH}_{2}^{*}+{CH}_{4(aq)}\to C_{2}H_{6}^{*}$	0.82	NC	0.52	NC	NC	NC	−0.03	1.60
11	${CH}_{2}^{*}+{CH}_{2}^{*}\to C_{2}H_{4}^{*}$	0.48	NC	0.13	NC	NC	NC	−0.61	1.48
12	${CH}_{2}^{*}+{CH}_{3}^{*}\to C_{2}H_{5}^{*}$	0.67	NC	0.72	NC	NC	NC	−0.13	1.36

NB: no barrier; NC: not calculated.

**Table 5:** The diffusion barriers for the movement of CH_2_ and CH_3_ adsorbed on M(111) surfaces from initial to final position in aqueous medium.

Adsorbates on M(111)	Diffusion Barrier (E_a_) (eV)			
	Co(111)	Ni(111)	Pd(111)	Pt(111)
${CH}_{2}^{*}$	0.48	0.46	1.03	0.67
${CH}_{3}^{*}$	0.04	0.50	0.28	0.65

**Table 6:** The rate constants (k) for the diffusion of CH_2_ and CH_3_ adsorbed, reactions 7, 11, and 12 on M(111) surfaces in aqueous medium.

Processes	Rate Constants (k) (M^−1^ s^−1^)			
	Co(111)	Ni(111)	Pd(111)	Pt(111)
Diffusion of ${CH}_{2}^{*}$	6.19 × 10^3^	1.68 × 10^4^	3.15 × 10^−6^	4.18
Diffusion of ${CH}_{3}^{*}$	9.65 × 10^11^	3.07 × 10^3^	2.25 × 10^7^	1.26 × 10^1^
Reaction 7 ($4{CH}_{3}^{*}$)	1.06 × 10^−9^	2.18 × 10^−18^	9.38 × 10^−3^	NC
Reaction 7 (5${CH}_{3}^{*}$)	1.69 × 10^3^	NC	1.03 × 10^1^	1.06 × 10^−11^
Reaction 7 (6${CH}_{3}^{*}$)	NC	NC	1.02 × 10^5^	4.76 × 10^−11^
Reaction 11 ($C_{2}H_{4}^{*}$)	NC	NC	NC	9.65 × 10^−14^
Reaction 12 ($C_{2}H_{5}^{*}$)	NC	NC	NC	1.06 × 10^−11^

NC: not calculated.

## Data Availability

The original contributions presented in this study are included in the article and Supplementary Materials. Further inquiries can be directed to the corresponding author(s).

## Supplementary Materials

The following supporting information can be downloaded at: https://www.mdpi.com/article/10.3390/molecules30153065/s1.
