# Supplementary material for: Investigation of the Adsorption and Reactions of Methyl Radicals on Transition Metal (M = Co, Ni, Pd, Pt) (111) Surfaces in Aqueous Suspensions
Source: Molecules. 2025 Jul 22;30(15):3065. doi: 10.3390/molecules30153065 (PMC12348179; doi:10.3390/molecules30153065)
Supplement: Supplementary file 1 [file molecules-30-03065-s001.zip › molecules-3765407-supplementary.pdf]

Supplementary Data

IInvestigation of the Adsorption and Reactions of Methyl Radicals on Transition Metal (M = Co, Ni, Pd, Pt) (111) Surfaces in Aqueous Suspensions

Pankaj Kumar [1], Dan Meyerstein [1,2,*], Amir Mizrahi [3] and Haya Kornweitz [1,*]

1 Chemical Sciences Department, The Radical Reactions Research Center, Ariel University, Ariel 4070000, Israel; pankajkumar121091@gmail.com

2 Chemistry Department, Ben-Gurion University, Beer-Sheva 8410501, Israel

3 Nuclear Research Centre Negev, Beer-Sheva 84190, Israel; amirmizrachi@gmail.com

* Correspondence: danm@ariel.ac.il (D.M.); hayak@ariel.ac.il (H.K.)

Figures

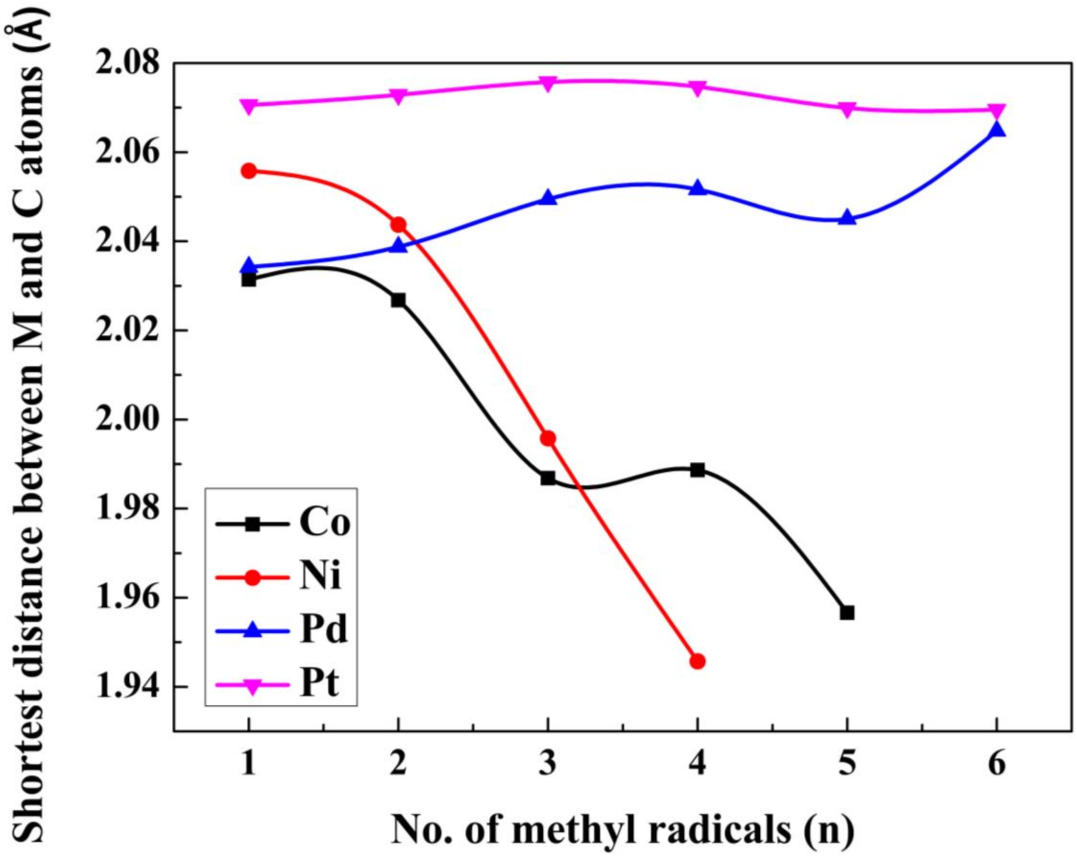

Figure S1: The shortest distance between M and carbon atoms (Å) under different surfaces coverage in aqueous phase.

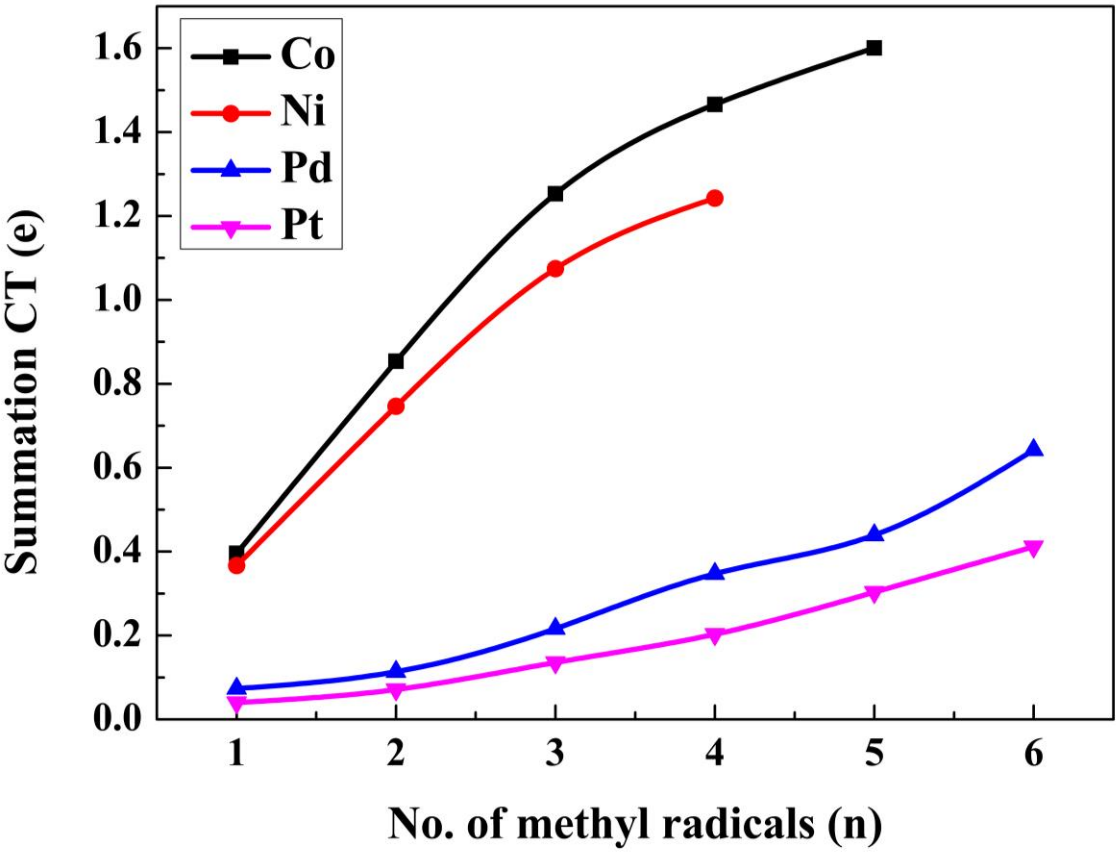

Figure S2: The summation of charge transfer (CT) of all methyl radicals on M(111) surfaces.

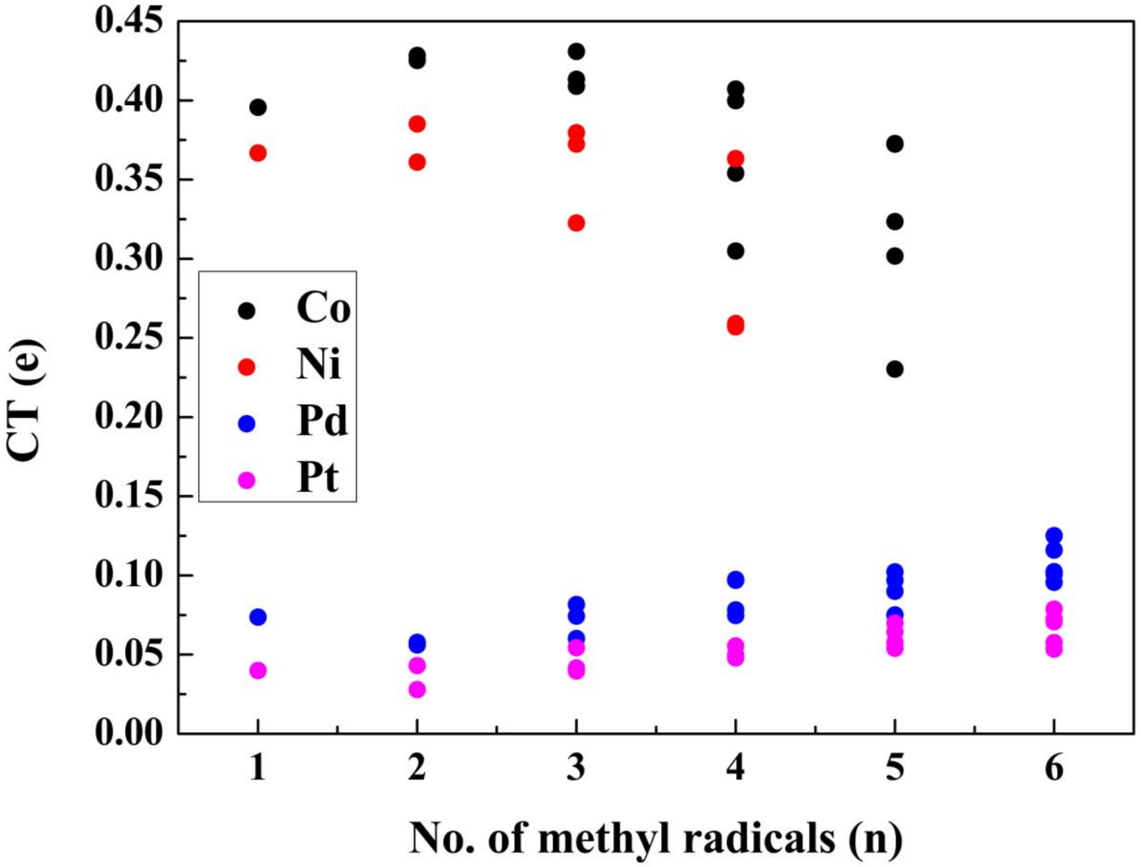

Figure S3: The charge transfer (CT) of every methyl radical (n) on M(111) surfaces.

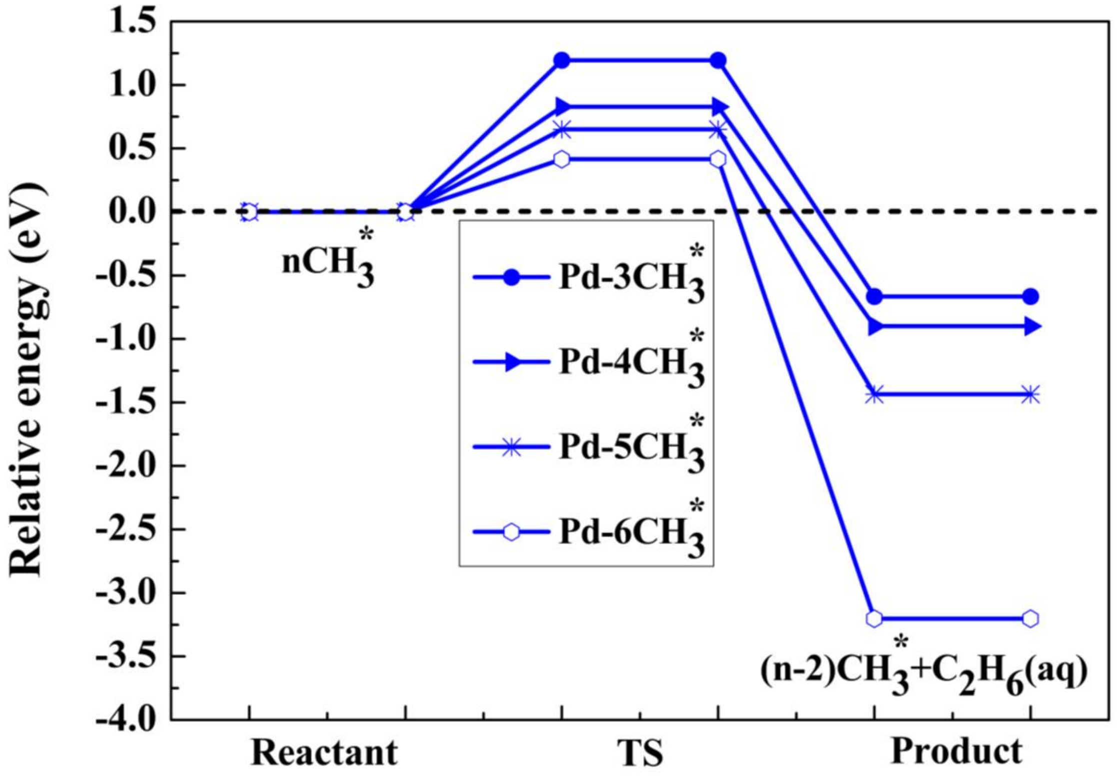

Figure S4: The E_a of 3 to 6CH_3 adsorbed on the Pd(111) surface for the evolution of ethane in aqueous medium.

Tables

Table S1: The evaluated ZPE (eV) values of the pristine metal surfaces and the adsorbed methyl radical and ethane at best adsorption sites in aqueous medium.

Metals	ZPE (eV)		
	Pristine surfaces	After CH_3 adsorption	After C_2H_6 adsorption
**Co**	1.72	2.56	3.63
**Ni**	1.62	2.51	3.57
**Pd**	1.12	2.06	3.08
**Pt**	1.09	2.04	3.04

Table S2: The calculated adsorption energies (E_{ads}) with ZPE (eV) values of the M(111) surfaces of the best adsorption of methyl radical and ethane in aqueous medium.

Metals	E_{ads} with ZPE (eV)	
	Best CH_3 adsorption	Best C_2H_6 adsorption
**Co**	-3.34	-0.55
**Ni**	-3.11	-0.75
**Pd**	-2.73	-0.68
**Pt**	-3.18	-0.99

Table S3: The estimated charge transfer (e) of a single methyl radical for different adsorption sites on M(111) surfaces.

Metals	Charge transfer (e)			
	**bridge**	**fcc**	**hcp**	**atop**
**Co**	0.40	0.40	0.40	0.22
**Ni**	0.35	0.37	0.36	0.20
**Pd**	0.06	0.05	0.05	0.07
**Pt**	0.03	0.04	0.03	0.04

Table S4: The estimated charge transfer (e) of ethane for different adsorption sites on M(111) surfaces.

Metals	Charge transfer (e)

	**bridge**	**fcc**	**hcp**	**atop**
**Co**	-0.003	0.001	-0.004	0.004
**Ni**	0.02	-0.01	-0.01	0.0003
**Pd**	0.01	-0.0004	-0.02	0.01
**Pt**	-0.03	-0.03	-0.04	-0.001

Table S5: All the optimized geometries (top view) of single methyl radical adsorbed on Co(111), Ni(111), Pd(111), and Pt(111) surfaces in aqueous phase.

**Metals**	**bridge**	**fcc**	**hcp**	**atop**
**Co**	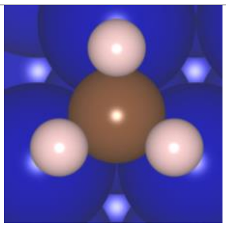	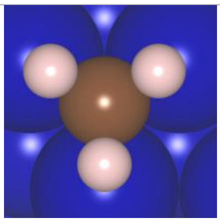	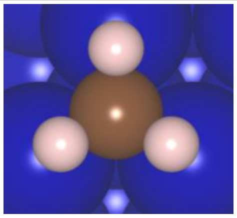	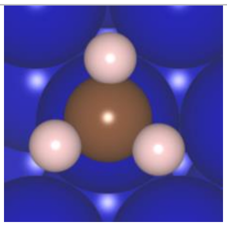
**Ni**	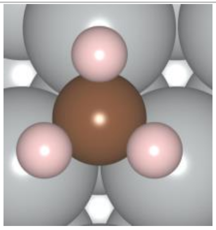	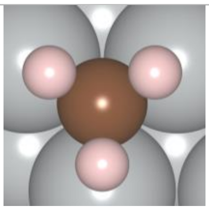	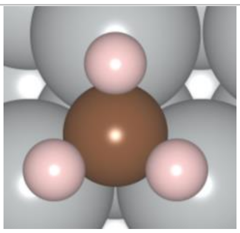	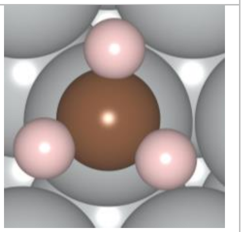
**Pd**	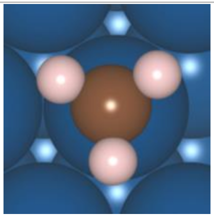	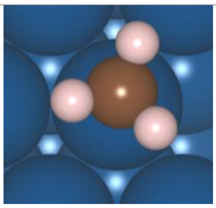	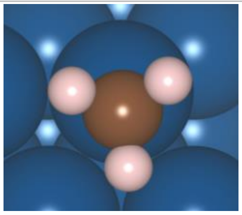	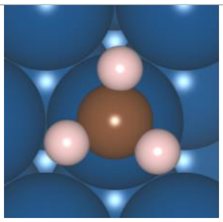
**Pt**	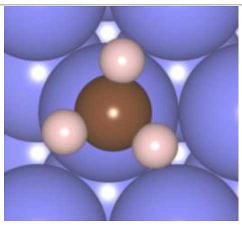	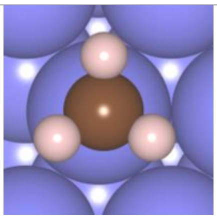	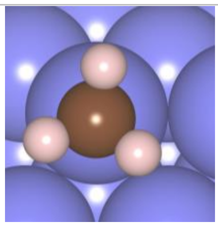	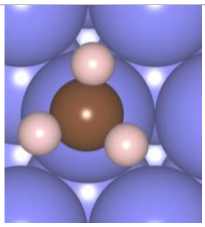

Table S6: All the optimized geometries (top view) of ethane adsorbed on Co(111), Ni(111), Pd(111) and Pt(111) surfaces.

Metals	bridge	fcc	hcp	atop
**Co**	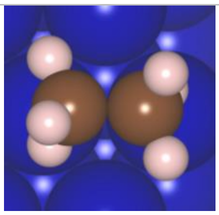	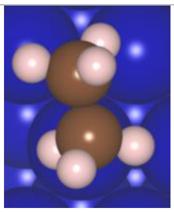	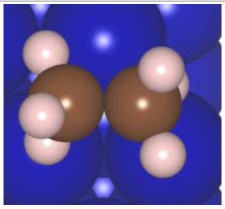	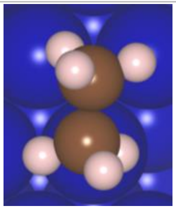
**Ni**	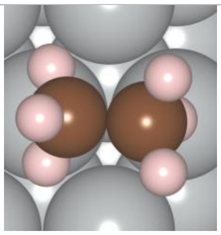	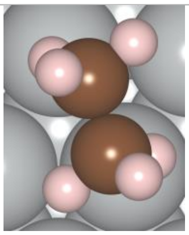	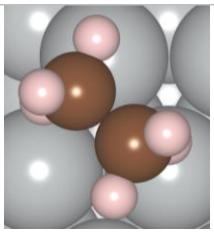	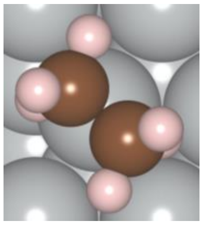
**Pd**	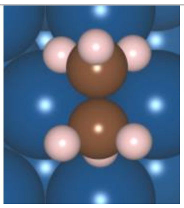	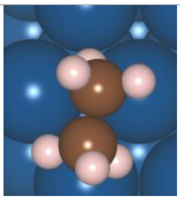	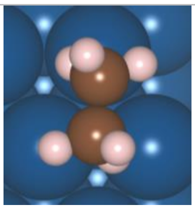	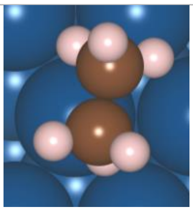
**Pt**	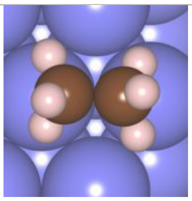	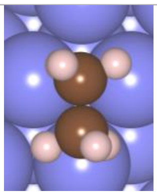	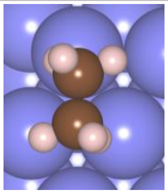	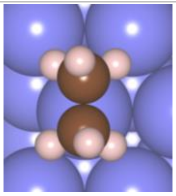

Table S7: The optimized geometries (top view) of n methyl radicals adsorbed on Co(111), Ni(111), Pd(111), and Pt(111) surfaces.

No. of methyl radicals	Co(111)	Ni(111)	Pd(111)	Pt(111)
1	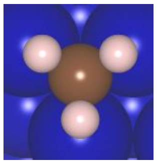	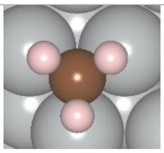	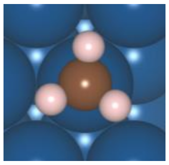	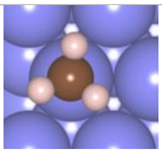

2	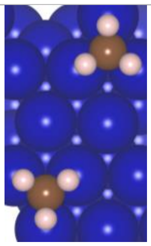	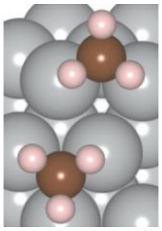	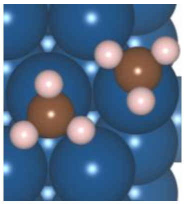	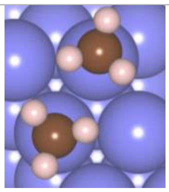
3	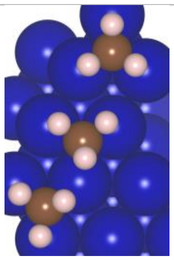	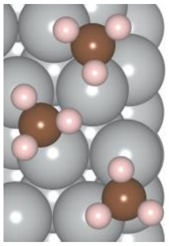	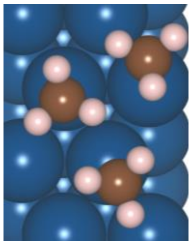	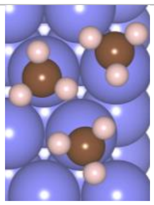
4	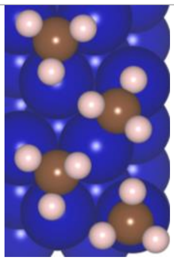	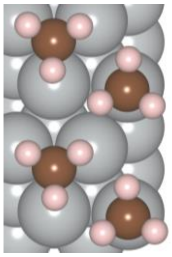	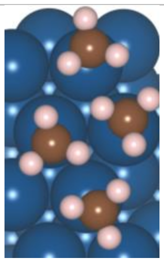	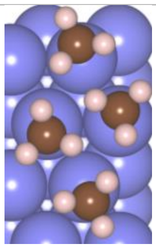
5	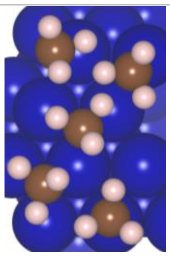	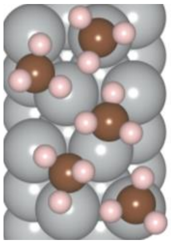	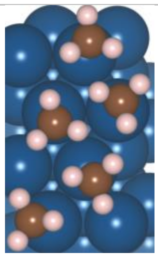	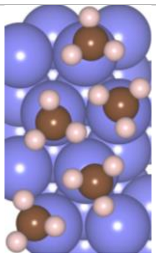
6	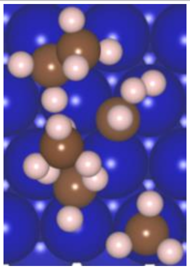	–	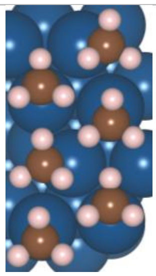	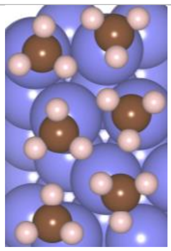

7	–	–	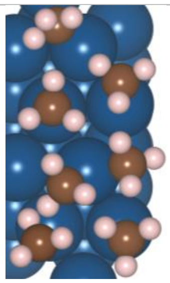	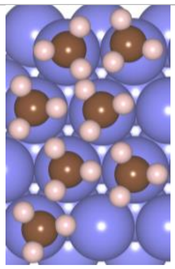

Table S8: Gibbs free energy of adsorption ($\Delta G_{CH_3(aq)}$) values on M(111) surfaces.

No. of methyl radicals	$\Delta G_{CH_3(aq)}$ (eV)			
	**Co(111)**	**Ni(111)**	**Pd(111)**	**Pt(111)**
1	-3.34	-3.11	-2.73	-3.18
2	-3.23	-3.00	-2.56	-3.08
3	-2.63	-2.51	-2.30	-2.79
4	-1.89	-2.23	-2.16	-2.59
5	-0.06	0.33	-1.75	-2.14
6	-4.76	–	-0.70	-1.19
7	–	–	-2.89	3.17

Table S9: The reaction free energy ($\Delta G^0_{C_2H_6(aq)}$) values on M(111) surfaces.

No. of methyl radicals	$\Delta G^0_{C_2H_6(aq)}$ (eV)			
	**Co(111)**	**Ni(111)**	**Pd(111)**	**Pt(111)**
2	1.67	1.20	0.34	1.35
3	0.95	0.60	-0.09	0.96

4	-0.39	-0.17	-0.49	0.47
5	-2.95	–	-1.04	-0.18
6	–	–	-2.50	-1.57

Table S10: The energy barriers (E_a) for the C_2H_6 evolution on M(111) surfaces at high coverage.

**Metals**	**No. of CH_3 on M(111)**	**E_a (eV)**
Co	4	1.24
	5	0.52
Ni	4	1.75
Pd	3	1.19
	4	0.83
	5	0.65
	6	0.41
Pt	5	1.36
	6	1.32

Table S11: The optimized geometries (top & side views) of reactant, transition state for reactions 8-13 on Co(111).

**Co(111) surface**	**Reactant**		**TS**	**Product**
	**Top view**	**Side view**		
$CH_3^* + CH_{3(aq)}^{\cdot} \rightarrow C_2H_6^*$	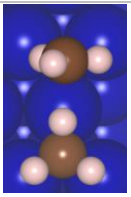	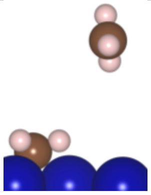	–	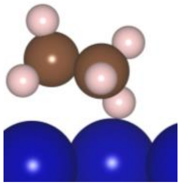

CH_3^* $+ CH_{3(aq)}$ $\rightarrow CH_2^*$ $+ CH_{4(aq)}$	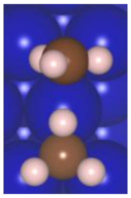	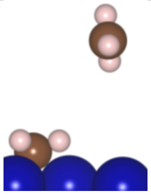	–	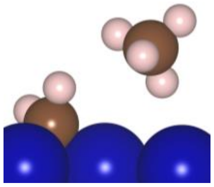
CH_2^* $+ CH_{4(aq)}$ $\rightarrow C_2H_6^*$	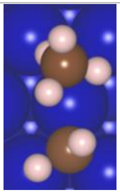	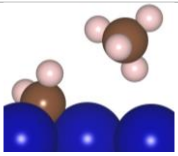	–	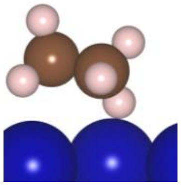
$CH_2^* + CH_2^*$ $\rightarrow C_2H_4^*$	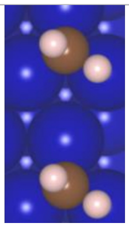	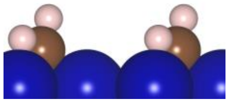	–	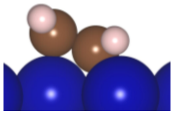
$CH_2^* + CH_3^*$ $\rightarrow C_2H_5^*$	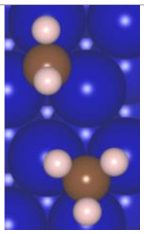	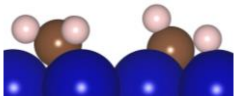	–	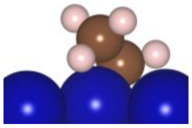
CH_2^* $+ \mathrm{CH}_{3(\mathrm{aq})}$ $\rightarrow \mathrm{C}_2\mathrm{H}_5^*$	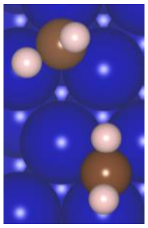	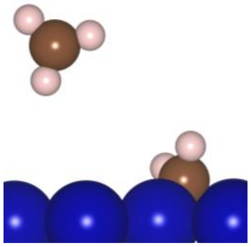	–	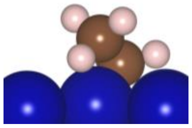
$\mathrm{C}_2\mathrm{H}_5^*$ $\rightarrow \mathrm{C}_2\mathrm{H}_4^*$ $+ H^*$	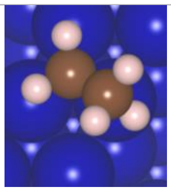	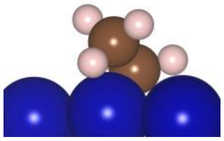	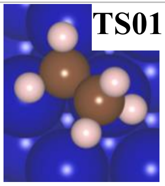	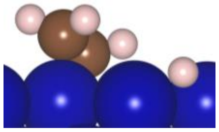

Table S12: The optimized geometries (top & side views) of reactant, transition state for reactions 8-13 on Ni(111).

Ni(111) surface	Reactant		TS	Product
	Top view	Side view		
$CH_3^* + CH_{3(aq)}^{\cdot}$ $\rightarrow C_2H_6^*$	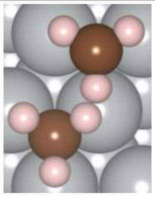	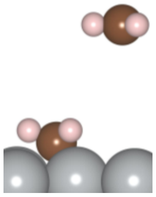	–	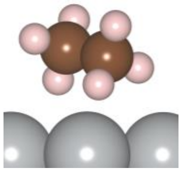
$CH_3^* + CH_{3(aq)}^{\cdot}$ $\rightarrow CH_2^*$ $+ CH_{4(aq)}$	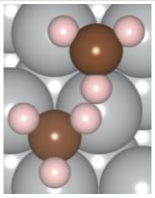	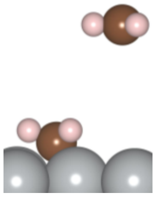	–	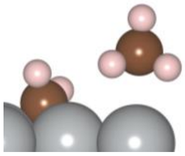
$CH_2^* + CH_{4(aq)}$ $\rightarrow C_2H_6^*$	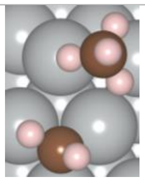	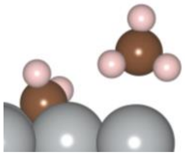	–	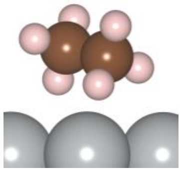
$CH_2^* + CH_2^*$ $= C_2H_4^*$	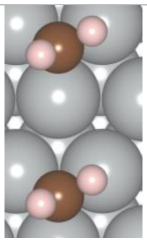	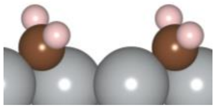	–	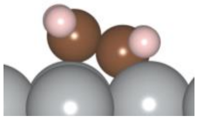
$CH_2^* + CH_3^*$ $\rightarrow C_2H_5^*$	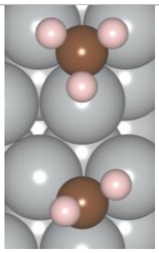	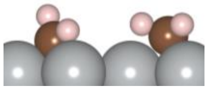	–	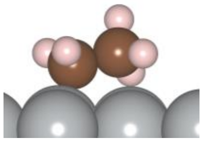

$CH_2^* + CH_{3(aq)}$ $\rightarrow C_2H_5^*$	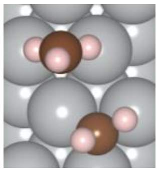	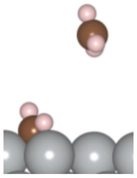	–	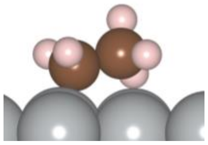
$C_2H_5^*$ $\rightarrow C_2H_4^* + H^*$	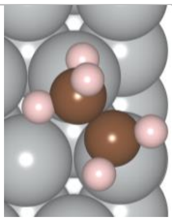	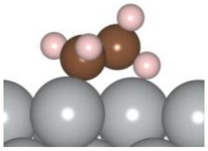	TS02 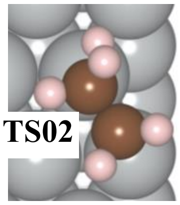	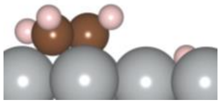

Table S13: The optimized geometries (top & side views) of reactant, transition state and product for reaction 8 on Pd(111).

Pd(111) surface	Reactant		TS	Product
	Top view	Side view		
$CH_3^* + CH_{3(aq)}^{\cdot}$ $\rightarrow C_2H_6^*$	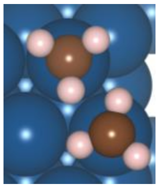	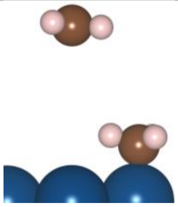	TS01 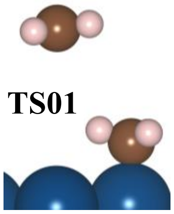	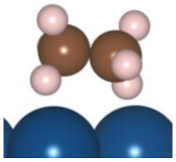

Table S14: The optimized geometries (top & side views) of reactant, transition state for reactions 8-14 on Pt(111).

Pt(111) surface	Reactant		TS	Product
	Top view	Side view		
CH_3^* $+ CH_{3(aq)}^{\cdot}$ $\rightarrow C_2H_6^*$	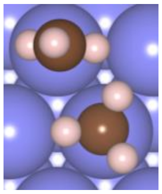	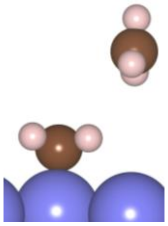	–	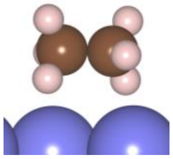

CH_3^* $+ CH_{3(aq)}$ $\rightarrow CH_2^*$ $+ CH_{4(aq)}$	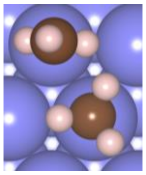	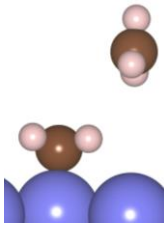	–	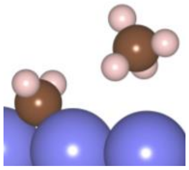
CH_2^* $+ CH_{4(aq)}$ $\rightarrow C_2H_6^*$	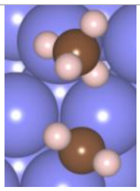	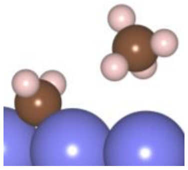	–	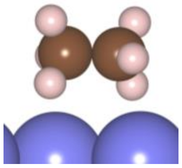
CH_2^* $+ CH_2^*$ $\rightarrow C_2H_4^*$	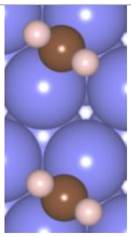	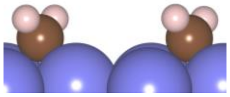	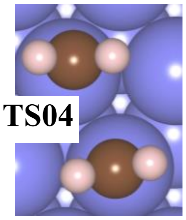	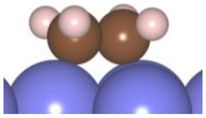
CH_2^* $+ CH_3^*$ $\rightarrow C_2H_5^*$	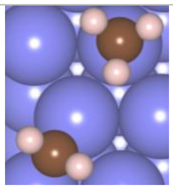	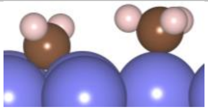	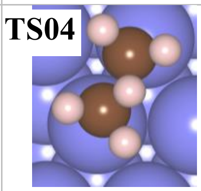	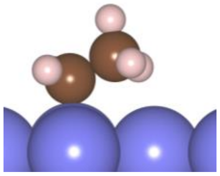
$C_2H_5^*$ $\rightarrow C_2H_4^*$ $+ H^*$	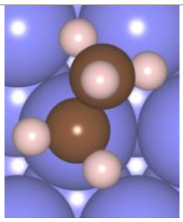	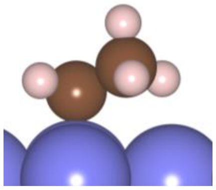	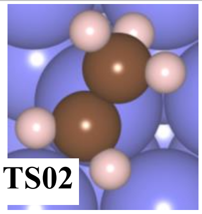	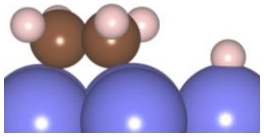
$C_2H_5^*$ $+ CH_3^*$ $\rightarrow C_2H_4^*$ $+ CH_{4(aq)}$	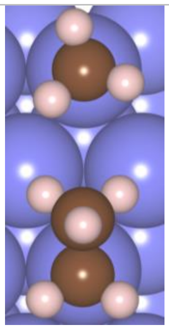	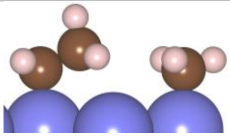	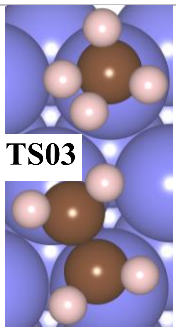	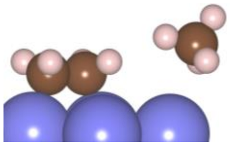

Table S15: The optimized geometries (top and side views) for the initial state, transition state, and final state, for the movement of adsorbed CH_2 and CH_3 on Co(111).

**Co(111) surface**	**Initial**		**TS**	**Final**
	**Top view**	**Side view**		
CH_2^*	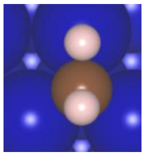	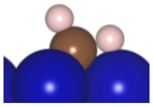	TS02 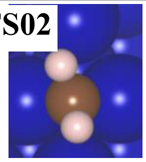	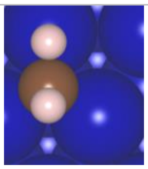
CH_3^*	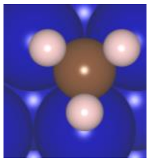	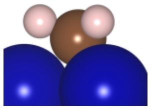	TS03 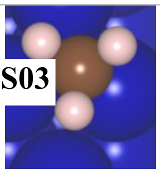	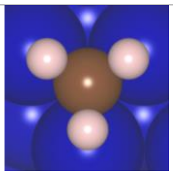
$CH_{3(aq)}^{\cdot} \rightarrow CH_3^*$	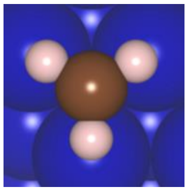	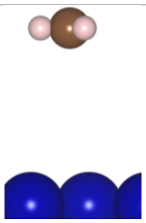	–	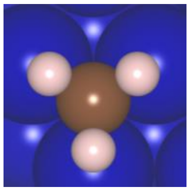

Table S16: The optimized geometries (top and side views) for the initial state, transition state, and final state, for the movement of adsorbed CH_2 and CH_3 on Ni(111).

**Ni(111) surface**	**Initial**		**TS**	**Final**
	**Top view**	**Side view**		
CH_2^*	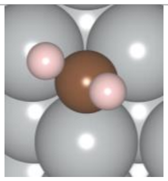	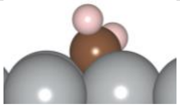	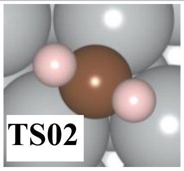 TS02	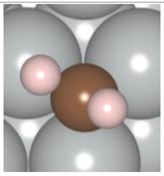
CH_3^*	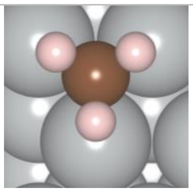	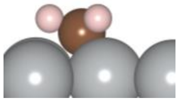	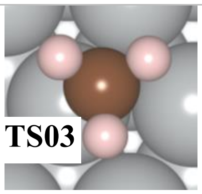 TS03	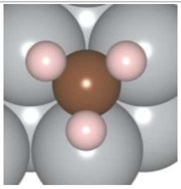

$CH_{3(aq)}^{\cdot} \rightarrow CH_3^*$	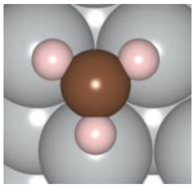	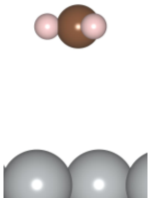	–	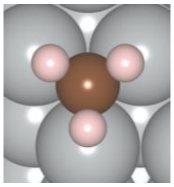

Table S17: The optimized geometries (top and side views) for the initial state, transition state, and final state, for the movement of adsorbed CH_2 and CH_3 on Pd(111).

**Pd(111) surface**	**Initial**		**TS**	**Final**
	**Top view**	**Side view**		
CH_2^*	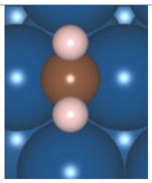	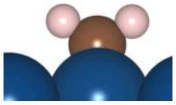	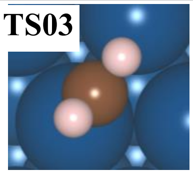	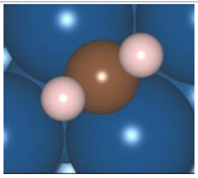
CH_3^*	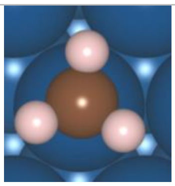	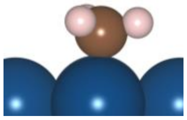	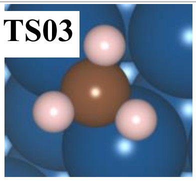	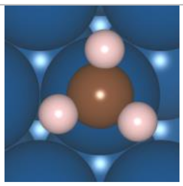
$CH_{3(aq)}^{\cdot} \rightarrow CH_3^*$	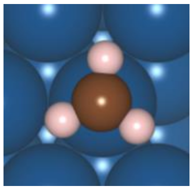	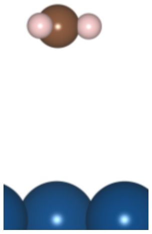	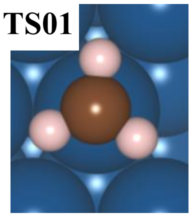	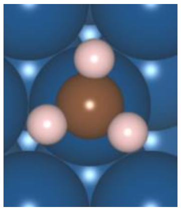

Table S18: The optimized geometries (top and side views) for the initial state, transition state, and final state, for the movement of adsorbed CH_2 and CH_3 on Pt(111).

**Pt(111) surface**	**Initial**		**TS**	**Final**
	**Top view**	**Side view**		

CH_2^*	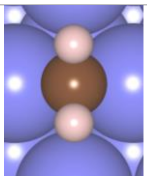	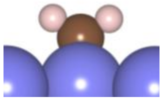	TS03 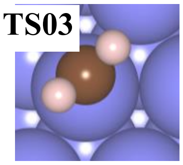	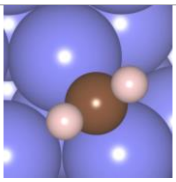
CH_3^*	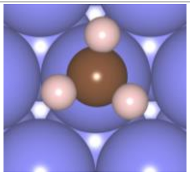	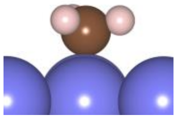	TS03 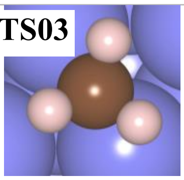	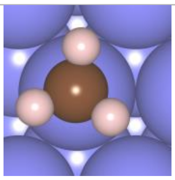
$CH^{\cdot}_{3(aq)} \rightarrow CH_3^*$	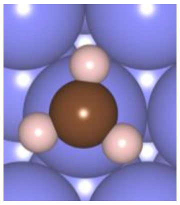	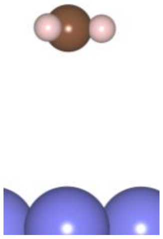	–	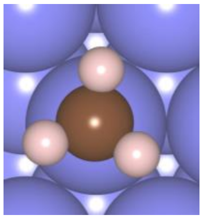
